# Our Academy1Presidential address delivered before the Buffalo Academy of Medicine June 11, 1907.

**Published:** 1907-09

**Authors:** Charles Sherman Jewett


### BUFFALO MEDICAL JOURNAL.

VOL. LXIII. SEPTEMBER, 1907. No. 2

### ORIGINAL COMMUNICATIONS.

### "Our Academy" [1]

BY CHARLES SHERMAN JEWETT, Ph.B., M.D.

IN casting about for a suitable subject for this address, I have gradually arrived at the conclusion that I can select none more pertinent than that of our Academy itself. This conclusion is based upon two reasons:—first, that our organisation is less effective, less influential and less successful than it should be; second, that we can and should make this Academy a power for good to the profession and to the public of Buffalo, a center for medical thought in our city, and a real aid to each of its members in the pursuit of his or her professional aims.

Our county society serves a useful purpose and we should all support it, but, until such time as the city of Buffalo is parted from the towns and erected into a separate county, the county society is unlikely to invade the field of the Academy's activities.

The various private medical clubs are admirable in their way, but, from their very organic structure, each must confine their benefits to a relatively small group of men or of women.

In my opinion this Academy should, within its own territory, be as broadly democratic in membership as is the county society. It should seek to serve the whole profession in our city. According to the new directory of the American Medical Association, Buffalo contains about six hundred and fifty physicians. Of this number probably about four hundred are eligible for fellowship in the Academy. Our resident fellowship is now 240. In other words, if we are to be a broadly representative body, we should nearly double our membership.

How are we to accomplish this? My answer would be,—by making fellowship in this Academy so valuable and so attractive that no intelligent practitioner who is eligible can afford to remain outside the fold. This sounds simple, but how is it to be brought

1. Presidential address delivered before the Buffalo Academy of Medicine June 11, 1907.

about? It seems to me that there are various lines along which we may work steadily and confidently toward this goal, and it is my purpose here to indicate several of those lines.

First, of course, the programs of our meetings should be made as valuable and as attractive as possible. This has always been done in a measure, and, during the season now closing, many of our programs have been of the highest merit. Along this line there is no need of any revolutionary change. Yet, with each new program, we may very properly seek to equal or excel the best in our past record. In this connection there comes up the annual discussion of the relative desirability of papers from our own members, and from distinguished men from other cities. To me it seems clear that there is plenty of room for both features: the ideal program would seem to be one which combines the best work of our own members with everything of real importance and value which we can secure from outside sources.

Second, we should have a bright, attractive and accessible meeting place. The gloom and ugliness of this hall are enough to sadden the members of any public gathering, and many of us believe that they have a distinct effect in preventing that full attendance which our meetings merit.

Third, we should endeavor further to promote social intercourse and good fellowship among our fellows. Closer personal acquaintance will do much in removing prejudice, correcting misconceptions, and in demonstrating to us that our professional colleagues are a far finer body of women and men than we have believed them to be. Such improved personal relations can not fail to increase our respect for one another, and thereby to enable us to work better together, both for the good of our own profession, and also for that of the community. In other words, if we knew and understood each other better we could unitedly exert a far greater influence in those public matters with which, of right, we ought to concern ourselves. The inauguration of social sessions with collations after our stated meetings has, I believe, done much to draw us together personally. Later, I hope to indicate other possible means for furthering this good end.

Fourth, the organisation of a well stocked reading room would greatly enhance the value of fellowship in this body. All of us, at times, desire access to the files of various medical periodicals to which we have no desire to subscribe. By united action we may easily have almost all of the valuable journals freely available by all our members. Such a reading room could not fail to increase our familiarity with the literature of our profession, while, incidentally, it would save us the cost of some of our less read journals. I do not overlook the admirable list of journals and the hospitality so freely offered us by the University of Buffalo, but, while appreciating these advantages and courtesies, I feel that the time has arrived when the medical men of our city should own and control their own reading-room. By a system of reciprocal courtesies between us and the university the values of both reading rooms might reach their maximum.

Fifth, at the earliest possible moment we should begin the formation of an Academy library. One of our fellows has recently written at some length upon this subject; I bring it up here because I firmly believe that we should have such a library in Buffalo, that we can have it, and that this Academy is the proper body to organise and administer it.

I doubt if any of our fellows will question the desirability of the various projects which I have indicated, although many of them will doubt their feasibility.

I am convinced that the time is ripe for the inauguration of a new era in the history of this Academy. As I have said to you before, I believe that the first step should be for us to acquire a home of our own.

This project has been canvassed most exhaustively by a special committee during the past year. While that committee has made and will make no definite report, I may say for its members that they are convinced of the desirability and practicability of the project, provided only that two questions can be answered in the affirmative—namely, do the fellows of the Academy wish to undertake such a project? and, second, are they willing to give it their hearty and united support? These are the questions which we should be glad to have discussed and answered.

Our permanent fund now amounts to $4,638.65.

Our income is about $1,012.00 annually, and increases with our membership.

We know that many of our fellows are ready and willing to subscribe to a building fund. We believe that some of our wealthy laymen would be willing to subscribe to such a fund if satisfied that our project would inure to the good of the profession and of the community. In addition to the subscriptions mentioned, we believe that many of our fellows would be willing to take second mortgage bonds of small denominations and at a moderate rate of interest.

If we are right in these beliefs, it would seem a simple matter to secure a central and accessible site. Upon this site, by alteration of existing buildings, or by new construction, we could promptly provide a suitable meeting room, a reading room, committee rooms, janitor's quarters and toilet facilities. Space should also be provided for such expansion as the future may show to be desirable. In particular, provision should be made for the erection of a fire proof library stack room.

Other points are less clear. It has been proposed that our building should have stores on the ground floor, also that our meeting hall might be rented for various gatherings when not required for our own use. On the other hand, it is quite possible that we might secure a charter as a scientific and literary institution which would exempt our property from taxation, provided that property were used exclusively for our own purposes.

Our investigations have led us to the belief that a site and building suitable for our present purposes and providing ample room for future expansion can be secured at a minimum price of $10,000 with $5,000 down, and the remainder on a mortgage. Of course, we could spend much more if we could get it.

Fellows of the Academy, for years I have longed to see this organisation in a home of its own, with facilities which should be adequate to its needs. I believe that the time has come when we may quite reasonably consider the advisability and practicability of some radical forward step. That step may be something entirely different from the plan outlined above, but I am firmly of the opinion that something definite should be undertaken to enhance the importance of the Academy and to render fellowship in it increasingly attractive.

The possession of a home for the Academy would enable us to have the sort of a meeting room we need, it would provide greatly increased opportunities for social intercourse, it would enable us at once to start our reading-room, and it would place us in a position where we could reasonably and hopefully plan for the organisation of our library. While thus meeting the indications which I have already pointed out, it would, I am sure, do far more.

It would give to each fellow a greater pride and a greater interest in the Academy. The institution would appear more dignified and substantial not only to its own members, but also to the laity, and this change would make it far easier for us to exert that influence which our united profession should possess in this community.

I appreciate, of course, that our prosperity and influence can not, and should not rest upon the amount of our possessions, nevertheless, I am confident that a scientific society provided with suitable quarters and with proper tools for its work can accomplish far more than one which has neither place nor equipment.

To me, however, the great argument is that the possession of a home and of proper facilities would unquestionably improve the *esprit* of our membership, that it would increase our interest in our own work and in that of our colleagues, and, above all, that it would inevitably make for better medical and surgical knowledge and practice, and hence, necessarily, for the good of the whole community.

The object seems worthy, the means within our grasp. The project should not be undertaken thoughtlessly. It is, I believe, worthy of serious consideration.

In all that I have said there is no implied criticism of the Academy's past nor of its present. My sole object has been to point out certain possibilities which the future may have in store for us. I am an optimist. I believe that our organisation is destined to enjoy increasing prosperity and usefulness whether it proceed along the old and well-tried lines, or whether it undertake something more pretentious.

We can not, however, remain stationary. We must advance or retrogress. Our past has been worthy; may we not plan for a future of greater accomplishments, and of enhanced usefulness?

In conclusion, I wish to thank you, the fellows of the Academy, for the honor of having been chosen as your president, and especially for the kindly way in which you have overlooked my many short-comings as presiding officer and executive.

To the officers of the Academy and of its sections, and to the members of its various committees I wish to express my sincere appreciation of their loyal and efficient service for the Academy.

To my successor I can offer no better wish than that he may find the new council as faithful, resourceful and effective a body as that over which it has been my privilege to preside.

892 Main Street.

